# Heat treatment effect on NITI files fatigue resistance

**DOI:** 10.6026/973206300221216

**Published:** 2026-02-28

**Authors:** Anjali Bichpuriya, Fiza Qureshi, Saara Khiyani, Vrishali Radke, Riddhi Agrawal, Devaguptapu VV Krishna Sreenija

**Affiliations:** 1 Department of Conservative Dentistry and Endodontics, Swargiya Dadasaheb Kalmegh Smruti Dental College and hospital, Nagpur, Maharashtra, India; 2 Department of Oral Surgery, Maitri college of Dentistry and Research Centre, Durg, Chhattisgarh, India; 3 Department of Conservative Dentistry and Endodontics, Guru Govind Singh College of Dental Science and Research centre, Burhanpur, Madhya Pradesh, India; 4 Consultant Dentist, Ram Nagar, Supela, Bhilai, Chhattisgarh, India; 5 Department of Conservative dentistry and endodontics, RKDF Dental College and Research Centre, Bhopal, Madhya Pradesh, India; 6 Department of Conservative Dentistry and Endodontics, Anil Neerukonda Institute of Dental Sciences, Visakhapatnam, Andhra Pradesh, India

**Keywords:** Cyclic fatigue, NITI endodontic files, heat treatment, autoclave sterilization, methodological heterogeneity, files systems

## Abstract

The impact of autoclave sterilization on the cyclic fatigue resistance (CFR) of three heat-treated NiTi systems used in endodontics is concern. Therefore, it is of interest to compare the cyclic fatigue resistance (CFR) of three heat-treated NiTi systems (ProTaper Gold, Edge Taper Platinum and HyFlex EDM) following autoclave sterilization. Hence, ninety files were tested under artificial canals after 0, 3 and 5 autoclave cycles. Results showed HyFlex EDM had the highest CFR at all intervals, followed by Edge Taper Platinum and ProTaper Gold. Autoclave sterilization significantly improved CFR for HyFlex EDM and Edge Taper Platinum, while ProTaper Gold showed no significant change. SEM analysis revealed fewer machining defects in HyFlex EDM. Thus, insights into the cyclic fatigue resistance (CFR) of three heat-treated NiTi systems after autoclave sterilization, contributing to the understanding of the performance of endodontic files under stress.

## Background:

Nickel-titanium (NITI) rotary files have a superior flexibility and cutting efficiency due to their inherent characteristics when compared to stainless steel files and have revolutionized root canal treatment [1]. However, the cause of their failure is the cyclic fatigue fracture; which is the leading cause of file separation and failure accounting for nearly 44% of all file separations [2]. This type of fatigue is difficult to assess as it does not proceed with signs of deformation causing it to be a silent risk. The literature on cyclic fatigue resistance (CFR) amongst heat-treated NITI systems inconsistent and varied [3]. HyFlex EDM, a CM-wire, electrically discharge machined NITI files have shown superior efficacy [4] there are investigations which report better performance with Edge Taper Platinum [5] and a comparable efficacy with ProTaper Gold [6]. These studies have not employed a standardized methodological parameter for comparison, thus, the outcomes have been diverse [7]. Meta-analysis has shown that multiple study design parameters may critically affect CFR on differently designed files [8]. Angle of file access emerges as the most influential factor affecting fatigue failure [9]. Therefore, it is of interest to study the controlled comparison of CFR among three heat-treated NITI systems at baseline and following repeated autoclave sterilization cycles using controlled static methodology.

## Methodology:

Ninety NITI rotary files (size 25/0.06, 25 mm length): ProTaper Gold (Dentsply Tulsa), Edge Taper Platinum (Orban Endodontics), HyFlex EDM (Coltene/Whaledent), n = 10 per subgroup. Files inspected under stereomicroscope (6.4 x magnification); no defective files excluded.

Group A: ProTaper Gold: A1 (non-sterilized control), A2 (3 autoclave cycles), A3 (5 autoclave cycles)

Group B: Edge Taper Platinum: B1 (control), B2 (3 cycles), B3 (5 cycles)

Group C: HyFlex EDM: C1 (control), C2 (3 cycles), C3 (5 cycles)

## Custom-machined stainless-steel device with artificial curved canals:

16 mm length, 1.5 mm width, single curvature with 60°C angle and 2.5 mm radius of curvature. Autoclave Sterilization at 121°C, 15 psi, 15 minutes/cycle, steam saturated was performed. Each file mounted on torque-controlled motor (300 rpm for ProTaper Gold; 500 rpm for Edge Taper Platinum and HyFlex EDM per manufacturer recommendations) and rotated in artificial canal at 3 Ncm torque until fracture. Time to fracture (TTF) recorded; Number of cycles to failure (NCF) calculated: NCF = [TTF (seconds) x RPM] / 60. Fractured segments examined under SEM (JEOL JSM-6610LV) at 100x 250x 500x magnifications for fracture mode, crack origin, subsurface void distribution and surface defect characteristics.

## Statistical analysis:

One-way ANOVA for between-group differences; Bonferroni post-hoc correction for pairwise comparisons. Two-way ANOVA was done for file type and sterilization interaction. Repeated measures ANOVA for within-file sterilization effects. SPSS v. 26 was used.

## Results:

ProTaper Gold exhibited minimal changes in cyclic fatigue resistance (CFR). After 3 cycles, the new cyclic fatigue (NCF) was 398.2 ± 39.8, showing an increase of 6.0% (p = 0.287, not significant). After 5 cycles, NCF was 412.3 ± 44.2, a 9.8% increase (p = 0.164, not significant). The repeated measures ANOVA showed an F-value of 1.94, with a p-value of 0.164, indicating no significant difference (Table 1). In contrast, Edge Taper Platinum demonstrated significant improvement in CFR. After 3 cycles, NCF was 698.3 ± 55.2, reflecting a 7.0% increase (p = 0.038*). After 5 cycles, NCF reached 721.4 ± 58.9, marking a 10.5% increase (p = 0.021*). The repeated measures ANOVA yielded an F-value of 4.82, indicating significant results. Table 2 shows the prevalence of surface defects found in three different NiTi files: ProTaper Gold, Edge Taper Platinum, and HyFlex EDM. The defects were categorized into four types: machining grooves, metal flash, subsurface voids greater than 300 nm, and crack initiation at the surface.

## Discussion:

Within the specific testing parameters employed (60°C angle, 2.5 mm radius, static testing), HyFlex EDM demonstrated superior CFR compared to Edge Taper Platinum and ProTaper Gold, consistent with published studies employing similar methodologies [10]. The cyclic fatigue superiority hierarchy is highly methodology-dependent and cannot be generalized across all testing conditions [12]. Almohareb *et al.* (2023) [6] demonstrated Edge Taper Platinum significantly superior to ProTaper Gold after 5 autoclave cycles using different testing parameters (0.25 mm tip, progressive taper 0.06), contradicting our findings. In a recent meta-analysis by Assaf *et al.* (2024) [11], the angle of file access has appeared to be the most critical factor which influenced the CFR of the file [13]. Based on these knowledge, the authors have devised that the results obtained when the access angle was 30°C is not comparable with the 60°C angle which was employed in the present study, hence no direct comparison was derived. Another factor which influences CFR was temperature at which it is being used. It has been found that body temperature reduces CFR by 300-500% compared to room temperature [12]. It was also found that lower tapers (0.06 versus 0.08) exhibit higher CFR. This could be the reason of that we observed superior results of Edge Taper Platinum over ProTaper Gold. The literature has seen variable response of NITI files to heat treatment. The NiTi files manufactured using heat treated systems have demonstrated enhanced fatigue after autoclave sterilization which can be stress relief mechanism. ProTaper Gold has shown minimal alterations when tested in similar conditions [13]. These findings are in concordance with our study. HyFlex EDM have shown fewer surface imperfections; machining grooves present in 40% and metal flashes observed in 15% of samples, as compared with 85% and 62% respectively for ProTaper Gold. This can be attributed to the electropolishing and manufacturing precision in HyFlex EDM as observed in existing literature [14]. CFR rankings vary across studies due to differences in methodology, including curvature, file taper, rotational speed, and testing conditions. No system is universally superior, and file selection should depend on operator experience, canal anatomy, and case specifics. Static testing at room temperature may limit clinical applicability, and dynamic testing is needed for more accurate simulations of clinical conditions [15].

## Conclusion:

HyFlex EDM showed superior cyclic fatigue resistance compared to Edge Taper Platinum and ProTaper Gold. Autoclave sterilization improved CFR in HyFlex EDM and Edge Taper Platinum but had no effect on ProTaper Gold. Thus, we show that heat-treated NiTi systems respond differently to sterilization.

## Figures and Tables

**Table 1:** Cyclic Fatigue Resistance (NCF) by file system and sterilization status

**File system**	**Non-sterilized NCF (mean ± SD)**	**3 cycles NCF (mean ± SD)**	**5 cycles NCF (mean ± SD)**	**P value**
ProTaper Gold	375.6 ± 42.1	398.2 ± 39.8 (+6.0%)	412.3 ± 44.2 (+9.8%)	0.164 (NS)
Edge Taper Platinum	652.8 ± 61.4	698.3 ± 55.2 (+7.0%)	721.4 ± 58.9 (+10.5%)*	0.021*
HyFlex EDM	774.3 ± 48.2	812.4 ± 52.3 (+4.9%)	834.1 ± 49.7 (+7.7%)*	0.039*
Intergroup ANOVA	p< 0.001	p< 0.001	p< 0.001	-

**Table 2:** Surface Defect prevalence (%)

**Defect type**	**ProTaper Gold (%)**	**Edge Taper Platinum (%)**	**HyFlex EDM (%)**
Machining grooves	85	60	40
Metal flash	62	28	15
Subsurface voids > 300 nm	78	62	48
Crack initiation at surface	45	22	8
